# Circulating cell-free DNA correlate to disease activity and treatment response of patients with radiographic axial spondyloarthritis

**DOI:** 10.1038/s41598-023-50543-0

**Published:** 2024-01-02

**Authors:** Yun Peng, Yuanhui Wu, Shiju Chen, Yuan Liu, Hongyan Qian, Yan He, Heqing Huang, Meimei Cai, Wen Liu, Guixiu Shi

**Affiliations:** 1 Department of Rheumatology and Clinical Immunology, The First Affiliated Hospital of Xiamen University, School of Medicine, Xiamen University, Xiamen, Fujian China; 2 Xiamen Municipal Clinical Research Center for Immune Diseases, Xiamen, Fujian China; 3 Xiamen Key Laboratory of Rheumatology and Clinical Immunology, Xiamen, Fujian China; 4 Department of Rheumatology and Clinical Immunology, Jiangxi Provincial People’s Hospital Affiliated of Nanchang University, Nanchang, Jiangxi China

**Keywords:** Biomarkers, Diseases, Rheumatology

## Abstract

Microdamage and its related inflammation contribute to the development of radiographic axial spondyloarthritis (r-axSpA). Inflammation and cell death in damaged tissues are associated with cell-free DNA (cfDNA) release. Here we investigated whether circulating cfDNA could be a potential biomarker for evaluating disease activity and treatment response in r-axSpA. Circulating cfDNA was detected in the discovery and validation cohort with 79 and 60 newly diagnosed r-axSpA patients respectively and 42 healthy controls using the Quant-iT PicoGreen dsDNA reagent and kit. As a result, cfDNA levels were significantly higher in r-axSpA patients compared with healthy controls in the discovery and validation cohort. Moreover, cfDNA levels were positively correlated with CRP, ASDAS-CRP and neutrophil counts. Additionally, non-steroid anti-inflammatory drugs (NSAIDs) combined with disease-modifying anti-rheumatic drugs or tumor necrosis factor inhibitors but not NSAIDs alone could reduce cfDNA levels. Moreover, a decrease of cfDNA levels after treatment was associated with an effective therapeutic response. Intriguingly, patients with higher levels of cfDNA at diagnosis responded better to combination therapy rather than NSAIDs. However, patients with lower levels of cfDNA displayed similar responses to combination or mono-NSAID treatment. In conclusion, circulating cfDNA levels showed a significant correlation with disease activity as well as treatment efficacy in patients with r-axSpA. Moreover, cfDNA at diagnosis might predict the response to different therapy. Consequently, cfDNA may serve as a useful biomarker of inflammation in r-axSpA.

## Introduction

Radiographic axial spondyloarthritis (r-axSpA) also known as ankylosing spondylitis (AS) is a chronic rheumatic disease with clinical features of inflammatory low back pain, enthesitis and axial skeleton progressive ossification, often accompanied by extra-articular manifestations such as psoriasis, anterior uveitis and inflammatory bowel disease, which can eventually lead to decreased spinal mobility and significant disability^1^. With the use of biologic agents that retard disease progression, early administration of therapy in non-responders could potentially improve long-term outcomes^2^. Unfortunately, acute phase reactants displayed limitations as measures of r-axSpA activity and treatment efficacy^3^. Hence, it is urgent to find a surrogate marker that reflects the inflammatory process and r-axSpA activity.

Cell-free DNA (cfDNA) is released into blood circulation as a result of damage or death of peripheral blood cells as well as organ tissues, which has been observed in several pathological conditions^4^. Recent studies had reported cfDNA as a surrogate marker to stratify patients, monitor the treatment response and predict disease progression, thus evaluating the prognostic potential of cfDNA for autoimmune diseases such as systemic lupus erythematosus (SLE) and rheumatoid arthritis (RA) as well as dermatomyositis (DM)^5–10^. Recently, accumulated evidence about environmental factors including mechanical stress and the microbiome has been established^11–14^. Microdamage caused by biomechanical stress initiates and perpetuates inflammation especially for innate immune responses^15^. As previously reported, cfDNA is mainly generated in the process of various types of cell death such as apoptosis, necroptosis and NETosis as well as pyroptosis in damaged tissues^4^. Moreover, cfDNA could be immunostimulatory provided it has access to intracellular DNA sensors^16,17^. Microdamage and its related inflammation under AS associated genetic conditions might result in higher cfDNA release in circulation. Therefore, it is reasonable to speculate that cfDNA may play a role in the pathogenesis of r-axSpA and subsequently might be a biomarker to assess r-axSpA activity. Here, we explored circulating cfDNA in patients with r-axSpA and its association with clinical disease activity and response to treatment.

## Methods

### Subjects and eligibility

Seventy-nine consecutive patients with r-axSpA were prospectively recruited from January 2018 to April 2019 as the discovery cohort, and the validation cohort with 60 newly diagnosed r-axSpA patients was enrolled from May 2020 to June 2021 in the Department of Rheumatology at the First Affiliated Hospital of Xiamen University. r-axSpA diagnosis was carried out according to the Assessment of SpondyloArthritis international Society (ASAS) classification criteria for axSpA (2009)^18^. Patients who were under 18 years old or had other diseases such as other autoimmune diseases, infections, hematologic diseases, malignancies, acute coronary syndrome, severe liver disease, renal function failure or treatment with corticosteroids within the last 3 months were excluded. Serum samples were harvested and stored at − 80 °C until use. Additionally, forty-two age- and sex-matched healthy Chinese volunteers were enrolled as healthy controls. Detailed clinical histories and laboratory examinations were recorded, including age, gender, duration of disease, C-reactive protein (CRP), erythrocyte sedimentation rate (ESR), blood routine examination and blood biochemistry. Blood samples were collected and stored at − 80 °C until use.

### Ethics

This study was approved by the ethics committee of The First Affiliated Hospital of Xiamen University and written informed consent was obtained from each individual. All studies were performed according to the Declaration of Helsinki.

### Treatment and clinical assessment

Sixty patients with r-axSpA in the validation cohort who underwent treatment with NSAIDs alone (n = 23), NSAIDs + DMARDs (n = 22) and NSAIDs + TNFi (n = 15) with a stable dose were followed up for at least 3 months. The evaluation of disease activity was performed according to the duration of early morning stiffness (EMS), tender and swollen joint counts, patient’s global assessment (PtGA, 0–10 scale), physician’s global assessment (PhGA, 0–10 scale), Bath Ankylosing Spondylitis Disease Activity Index (BASDAI), Ankylosing Spondylitis Disease Activity Score (ASDAS)-CRP and ASDAS-ESR. Disease activity at baseline was evaluated by ASDAS-CRP (≥ 1.3), ASDAS-ESR (≥ 1.3) and BASDAI (≥ 4) respectively. Responders were defined as the patients experienced a decline of ASDAS-CRP at least 1.1 after 3 months post-treatment while those failed to achieve the criteria were classified as non-responders^19^.

### Measurement of circulating cfDNA

Circulating cfDNA levels were evaluated at diagnosis in all 79 patients in the discovery cohort and 60 patients in the validation cohort, whereas only 51 patients were evaluated at both diagnosis and 3 months posttreatment in the validation cohort. Vacuum serum tubes were used to collect 3 ml of venous blood from all participants after 12 h of fasting. The tubes were centrifuged at 2500 rpm for 15 min to obtain the serum. Serum samples were frozen at − 20 °C within eight hours of collection and then transferred to a − 80 °C environment for long-term storage. Samples were thawed at 4 °C prior to assay performance. Serum circulating cfDNA was measured by using the Quant-iT PicoGreen dsDNA reagent and kit (Invitrogen, California, USA). TE buffer (10 mM Tris–HCl, 1 mM EDTA, pH 7.5), free of contaminating nucleic acids was used to dilute the Quant-iT PicoGreen reagent and DNA samples. Standard curves were repeated in each 96-well plate. Simply, PicoGreen, an ultrasensitive fluorescent nucleic acid stain for quantitating double-stranded DNA (dsDNA) that can detect and quantitate small amounts of DNA, was added to the serum, and a fluorescence microplate reader was used to quantify the DNA content with a filter setting of 485/535 nm excitation/emission wavelengths. Each sample was performed in duplicate in the same plate.

### Statistical analysis

The statistical analysis was carried out using GraphPad Prism 8.0 software (GraphPad Software, San Diego, CA, USA) and SPSS 22.0 (IBM Corporation, Armonk, NY, USA). The results were expressed as count (%) or median (range). The Mann–Whitney *U* test was used to compare the differences in continuous variables and Fisher’s exact test was used for categorical variables. Correlations were calculated using Spearman’s rank correlation test. The chi-square test was used to compare the frequencies between two groups of categorical variables. A P value < 0.05 was considered statistically significant. The following symbols were used: *P < 0.05; **P < 0.01; ***P < 0.001; ****P < 0.0001; and ns, not significant.

## Results

### Patients’characteristics

Seventy-nine de novo r-axSpA patients were enrolled in the discovery cohort, and 60 patients were enrolled in the validation cohort to evaluate cfDNA at diagnosis. The baseline demographic features including age (33 ± 7) and gender (30 males and 12 females) were similar between r-axSpA patients and healthy controls. The patients’ features were summarized in Table 1. Only one patient in discovery cohort experienced uveitis as the extra-articular manifestation, the rest patients in both cohorts showed no manifestations of other extra-articular involvement such as psoriasis, inflammatory bowel disease, cardiovascular, pulmonary or renal involvement. Active disease was identified with BASDAI (19%), ASDAS-CRP (66%) and ASDAS-ESR (72%) in the discovery cohort and BASDAI (5%), ASDAS-CRP (52%) and ASDAS-ESR (75%) in the validation cohort. Sixty patients in the validation cohort were followed up for at least 3 months and cfDNA detection was performed in 51 of these patients after initial therapy. Among the 60 patients, patients who received NSAIDs alone, NSAIDs + DMARDs, and NSAIDs + TNFi accounted for 38%, 37% and 25% respectively. The remission rate was 52% in the NSAIDs group and 55% in the NSAIDs + DMARDs group, while in the NSAIDs + TNFi group, 67% achieved remission.

**Table 1:** Demographic data and clinical characteristics of enrolled subjects.

Characteristics	Discovery cohort (N = 79)	Validation cohort (N = 60)
Age, mean (range) years	34.2 (21–66)	34.7 (19–59)
Gender, n (%)		
Male	60 (76)	46 (77)
Female	19 (24)	14 (23)
HLA-B27 positive rate, n (%)	74 (94)	55 (92)
Extra-articular involvement, n (%)	1 (1.3)	0 (0)
Disease duration, n (%)		
≤ 5 years	29 (37)	19 (32)
> 5 years	50 (63)	41 (68)
CRP, mean (range) mg/L	5.2 (0.04–25.3)	5.4 (0.1–22.2)
ESR, mean (range) mm/h	12.3 (2–61)	10.5 (2–37)
cfDNA, mean (range) ng/ml	226.9 (103–410)	246.0 (142–487)
BASDAI, (n)%		
Active	15 (19)	3 (5)
Inactive	64 (81)	57 (95)
ASDAS-CRP, (n)%		
Active	52 (66)	31 (52)
Inactive	27 (34)	29 (48)
ASDAS-ESR, (n)%		
Active	57 (72)	45 (75)
Inactive	22 (28)	15 (25)
PtGA, mean (range)	3.0 (1–8)	2.6 (0–9)
PhGA, mean (range)	2.3 (0–8)	2.7 (0–9)
Medications, n (%)		
NSAIDs	23 (29)	23 (38)
NSAIDs + DMARDs	27 (34)	22 (37)
NSAIDs + TNFi	29 (37)	15 (25)

Data are presented as count (%) or median (range). *CRP* C-reactive protein, *ESR* erythrocyte sedimentation rate, *cfDNA* cell-free DNA, *BASDAI* Bath Ankylosing Spondylitis Activity Disease Activity Index, *ASDAS* Ankylosing Spondylitis Disease Activity Score, *PtGA* Patient’s global assessment*, PhGA* Physician’s global assessment, *NSAIDs* non-steroid anti-inflammatory drugs, *DMARDs* disease-modifying anti-rheumatic drugs, *TNFi* tumor necrosis factor inhibitor.

### Higher levels of circulating cfDNA in patients with different subgroups of r-axSpA compared with healthy individuals

**Figure 1:**
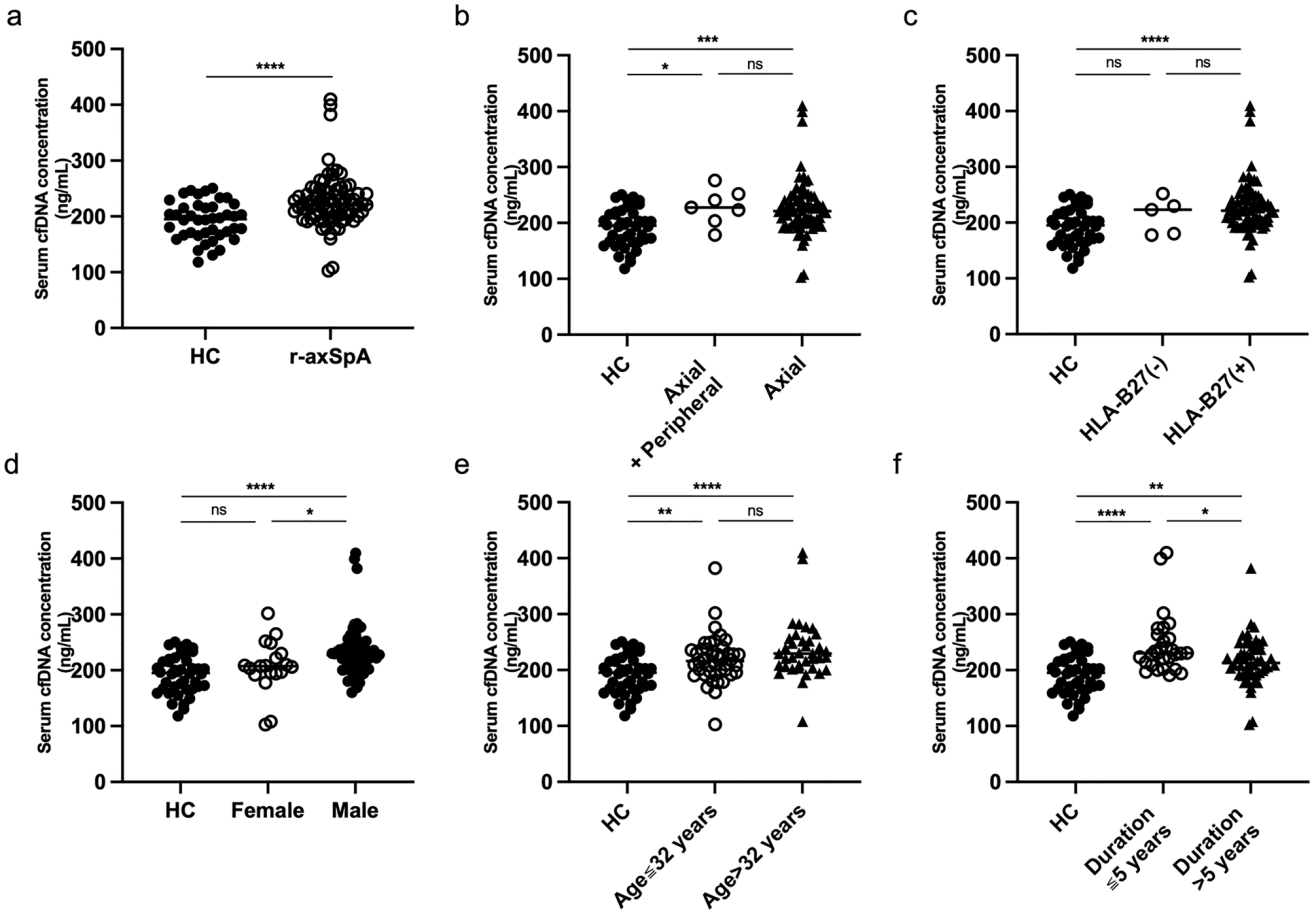
Higher cfDNA levels in patients with different subtypes of r-axSpA compared with healthy individuals. (**a**) Serum cfDNA levels were higher in r-axSpA patients than in healthy controls (HC). (**b**) Serum cfDNA levels in patients with or without peripheral joint involvement. (**c**) Serum cfDNA levels in patients with or without expression of HLA-B27. (**d**) Serum cfDNA levels were higher in men than in women. (**e**) Serum cfDNA levels in two groups of patients divided by median age of 32 years. (**f**) Serum cfDNA levels were significantly higher in patients with a short disease duration (≤ 5 years) than in patients with a long disease duration (> 5 years). Each data point represents a separate subject. Horizontal lines show the mean value of patients with r-axSpA and healthy controls. *P < 0.05, **P < 0.01, ***P < 0.001, ****P < 0.0001 and *ns* not significant.

To evaluate the circulating cfDNA in patients with r-axSpA, a Quant-iT PicoGreen assay was used to quantify serum cfDNA. As a result, compared with healthy controls, r-axSpA patients had significantly higher levels of cfDNA (227 ± 48 vs 192 ± 34 ng/ml, P < 0.0001) (Fig. 1a). Further subgroup analysis showed that the concentrations of cfDNA were comparable between patients with or without peripheral joint involvement (Fig. 1b) as well as patients with or without expression of HLA-B27 (Fig. 1c). Moreover, cfDNA levels were higher in men than in women, which is consistent with previous research reporting greater inflammation in men^20,21^ (Fig. 1d). We further divided the patients into two groups based on a median age of 32 years and found that cfDNA levels were similar in both groups (Fig. 1e). In addition, we further divided patients into two groups with disease duration ≤ 5 years and > 5 years and found that cfDNA levels were significantly higher in patients with a short disease duration than in patients with a long disease duration (Fig. 1f).

### The association of circulating cfDNA levels and disease activity in r-axSpA patients

**Figure 2:**
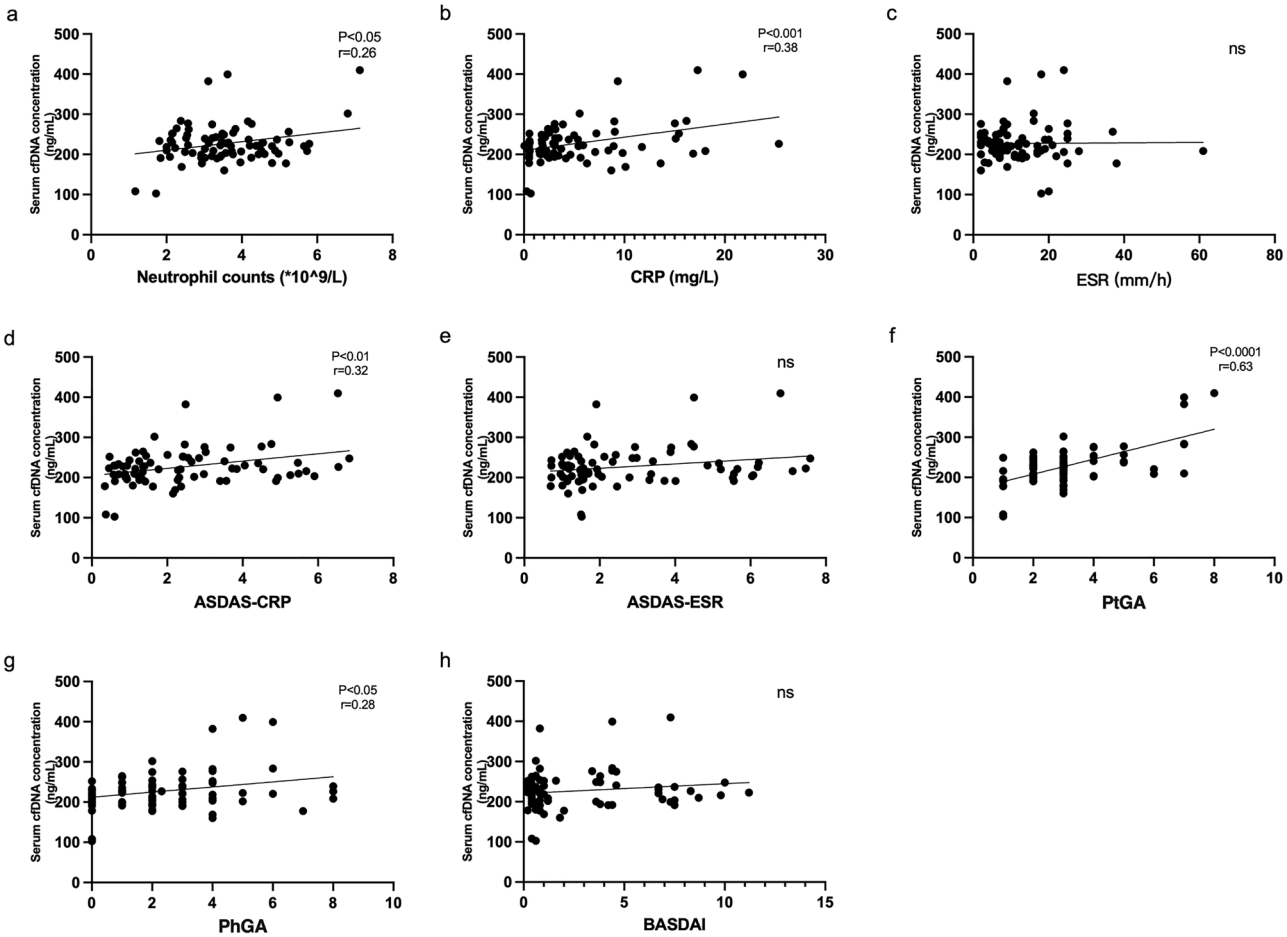
Serum cfDNA levels were associated with disease activity. (**a**–**h**) Correlations between the concentrations of cfDNA and (**a**) neutrophil counts, (**b**) C-reactive protein (CRP), (**c**) erythrocyte sedimentation rate (ESR), (**d**) ASDAS-CRP, (**e**) ASDAS-ESR, (**f**) patient’s global assessment (PtGA), (**g**) physician’s global assessment (PhGA), and (**h**) Bath Ankylosing Spondylitis Activity Disease Activity Index (BASDAI). Each data point represents a separate case.

Neutrophils are involved in NETosis and the generation of cfDNA and are also increased in inflammatory settings. Therefore, the correlation between cfDNA and neutrophils was evaluated. A significantly positive correlation between cfDNA concentrations and neutrophil counts was observed in patients with r-axSpA (Fig. 2a). CRP and ESR were the biomarkers for inflammation and were applied as biomarkers for disease activity in the ASDAS. As shown, cfDNA concentrations significantly correlated with CRP (Fig. 2b) rather than ESR (Fig. 2c). Similarly, cfDNA levels also displayed an obvious correlation with ASDAS-CRP (Fig. 2d) but not ASDAS-ESR (Fig. 2e). In addition, we further evaluated the association of cfDNA and disease activity assessed by clinical manifestations such as BASDAI and patient’s/physician’s global assessment. Accordingly, cfDNA levels also correlated with patient’s global assessment (Fig. 2f) and physician’s global assessment (Fig. 2g). However, BASDAI (Fig. 2h) was not associated with the concentration of cfDNA. These data suggest that cfDNA levels are associated with disease activity in r-axSpA patients.

### Dynamic alterations of circulating cfDNA levels in patients receiving initial therapy

**Figure 3:**
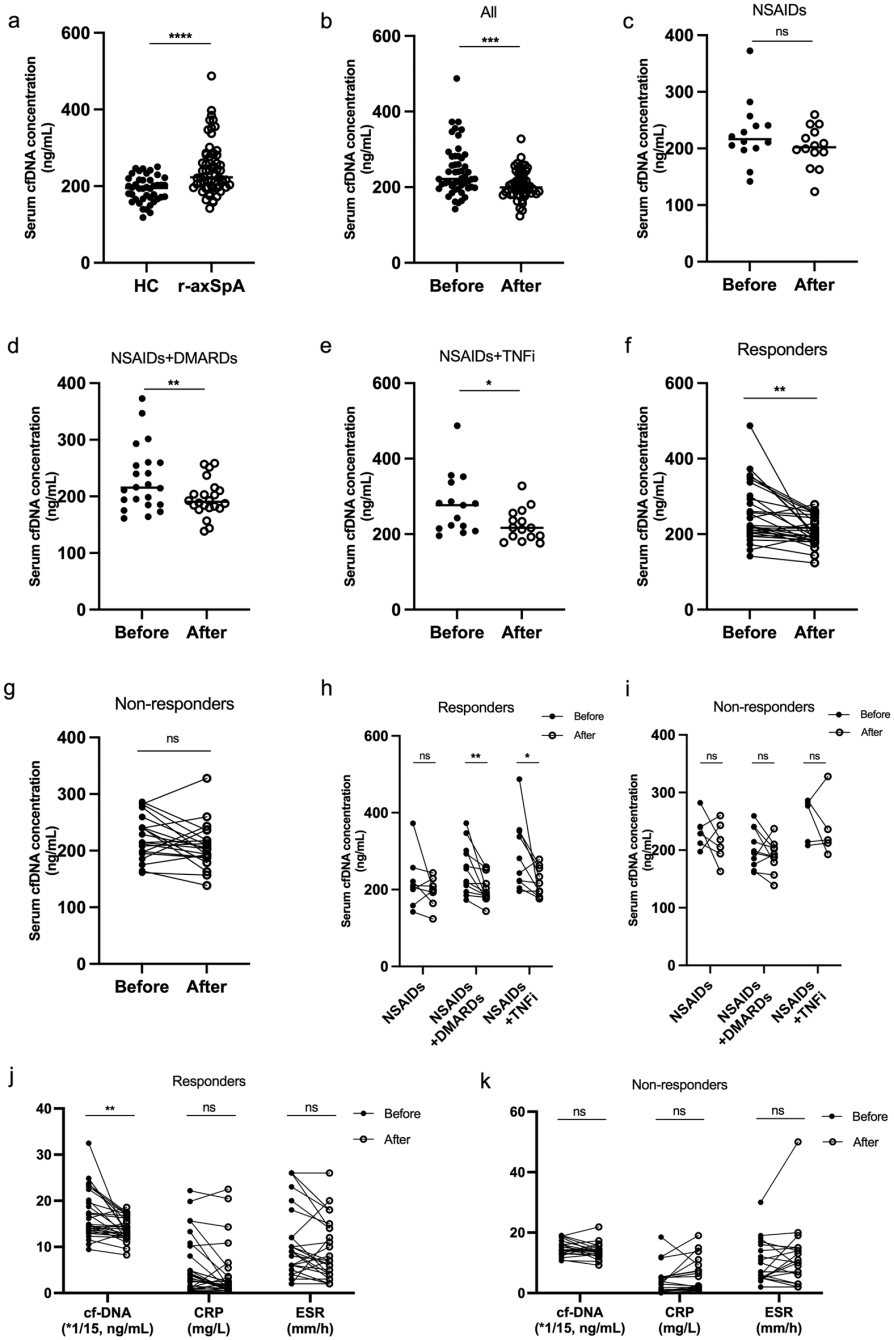
Serum cfDNA levels decreased with therapy in r-axSpA patients until 3 months from baseline. (**a**) Serum cfDNA levels were higher in the validation cohort at diagnosis than in healthy controls (HC). (**b**) Serum cfDNA levels decreased in all 51 patients after treatment. (**c**) Serum cfDNA levels in patients treated with NSAIDs alone after treatment. (**d**) Serum cfDNA levels decreased in patients treated with NSAIDs + DMARDs after treatment. (**e**) Serum cfDNA levels decreased in patients treated with NSAIDs + TNFi after treatment. (**f**) Serum cfDNA levels decreased in the responders group after treatment. (**g**) cfDNA levels in the non-responders group were not significantly reduced after treatment. (**h**) cfDNA levels fell with treatment containing DMARDs or TNFi but not NSAIDs alone in the responders group. (**i**) cfDNA levels in the non-responders group were not significantly reduced in any of the treatment groups. (**j**–**k**) Serum levels of cfDNA, CRP and ESR after treatment in the responders group and the non-responders group. Each data point represents a separate case. *P < 0.05, **P < 0.01, ***P < 0.001, ****P < 0.0001 and *ns* not significant.

To validate the role of cfDNA in r-axSpA, a validation cohort with 60 r-axSpA patients was enrolled. Consistent with the discovery cohort, patients in the validation cohort also displayed higher levels of cfDNA than healthy controls (Fig. 3a). Meanwhile, the dynamic alterations of cfDNA and its correlation to disease status or treatment response were evaluated in 51 of 60 patients. The cfDNA concentrations were significantly decreased until 3 months from baseline (243 ± 66 vs 206 ± 38 ng/ml, P < 0.0001, Fig. 3b). As shown in Fig. 3, cfDNA levels were significantly reduced in patients treated with NSAIDs + DMARDs (230 ± 56 vs 197 ± 32 ng/ml, P < 0.05, Fig. 3d) and NSAIDs + TNFi (277 ± 76 vs 223 ± 41 ng/ml, P < 0.05, Fig. 3e) groups, but not in patients treated with NSAIDs alone (Fig. 3c). Additionally, the correlation between cfDNA concentrations and clinical responses was further evaluated. As a result, cfDNA concentrations in the responders group declined significantly from baseline until 3 months (260 ± 73 vs 211 ± 32 ng/ml, P < 0.01, Fig. 3f), whereas those non-responders did not (Fig. 3g). Moreover, cfDNA levels fell with treatment containing DMARDs or TNFi but not NSAIDs alone in responders (Fig. 3h). However, cfDNA levels were comparable at diagnosis and after treatment in non-responders regardless of treatment (Fig. 3i). CRP and ESR were also evaluated in patients receiving initial therapy. Unlike cfDNA, the levels of CRP and ESR did not significantly change after treatment in both responders and non-responders (Fig. 3j,k).

### cfDNA levels at diagnosis predict the response to the therapeutics

**Figure 4:**
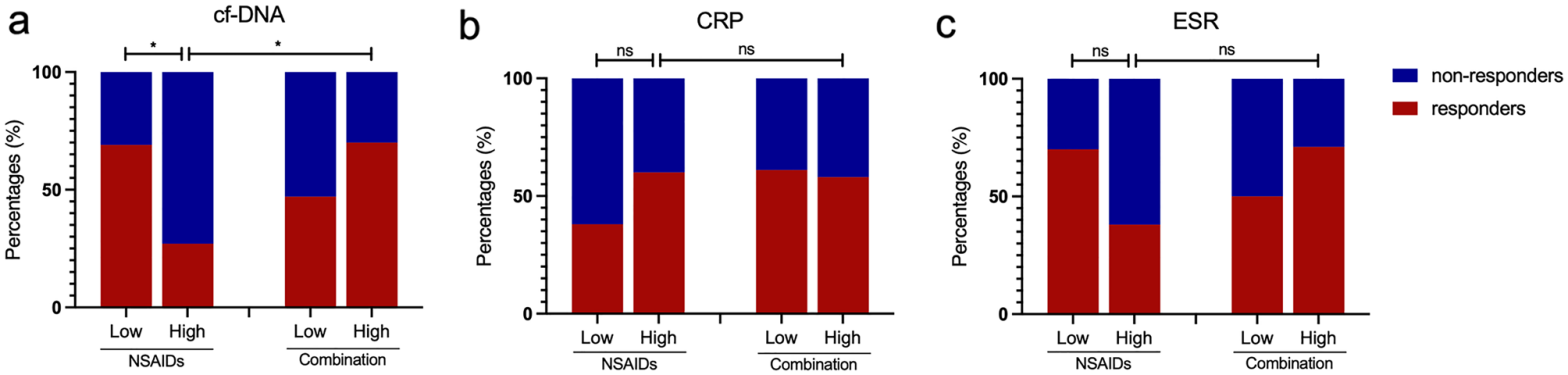
Response rate in patients in the NSAIDs group and the combination group (NSAIDs combined with DMARDs or TNFi). (**a**–**c**) Response rate in patients in the NSAIDs group and the combination group according to the median values of cfDNA, CRP and ESR. *P < 0.05 and *ns* not significant.

Although CRP and ESR and other biomarkers are used to reflect disease activity, the biomarkers reflecting response to therapeutic drugs remain to be explored. We evaluated the role of cfDNA at diagnosis in predicting response to the initial treatment. Sixty patients receiving evaluation of clinical response were divided into high and low groups based on the median values of cfDNA, CRP and ESR at diagnosis. Patients with low cfDNA levels showed similar response rates in the NSAIDs group and the combination group (NSAIDs combined with DMARDs or TNFi). However, patients with higher cfDNA levels displayed a significantly poorer response to NSAIDs. Intriguingly, combination therapy could reverse the poor response to NSAIDs in patients with higher cfDNA (Fig. 4a). Nevertheless, patients with higher CRP or ESR at diagnosis showed similar responses to NSAIDs and combination therapy. Additionally, lower levels of CRP or ESR at diagnosis did not predict a good response to NSAIDs (Fig. 4b,c). Therefore, cfDNA might be linked to sensitivity to therapeutics, which might guide and optimize the clinical decision on the initial therapy.

## Discussion

In the present study, r-axSpA patients showed significantly higher cfDNA levels compared with healthy controls. cfDNA levels were positively correlated with disease activity. Furthermore, a decrease in cfDNA after treatment was associated with a favorable clinical response. Moreover, cfDNA at diagnosis might predict response to treatment. Consequently, these data indicated that circulating cfDNA may serve as a promising biomarker of r-axSpA activity and treatment response.

Endogenous DNA is primarily found intracellularly in nuclei and mitochondria. However, cell death including apoptosis, necrosis, NETosis and pryptosis, could trigger the active release of cfDNA^4^. Despite higher cfDNA in r-axSpA patients, the source of cfDNA remains unknown. Rationally, microdamage by recurrent mechanical stress in the early stage and later bone destruction might serve as a source of dead cells in r-axSpA. Meanwhile, pyroptosis of neighboring cells around innate immune cells induced by inflammatory settings of AS was also reported^22^. Thus, both cell death types might contribute to higher cfDNA in r-axSpA. Additionally, NETosis is also considered a main source of cfDNA, which has been reported in SLE, DM and RA^6–9^. Our data also showed that cfDNA levels were positively correlated with neutrophil counts in r-axSpA patients, which may suggest that NETosis is one of the sources of cfDNA in r-axSpA patients.

Additionally, inflammation could also promote generation of NETosis and pyroptosis and subsequently contribute to release of cfDNA. Active disease of r-axSpA is closely associated with increased inflammation. Consistent with SLE and RA, cfDNA levels could positively correlate with laboratory-based disease activity such as CRP and ASDAS-CRP. Ho Yin Chung et al. demonstrated that early SpA display more intensive inflammation^23^. Early SpA is generally considered as disease duration less than 5 years^24^. Although patients in the current study showed radiographic manifestations, significant higher cfDNA levels were observed in patients with disease duration no more than 5 years. Therefore, even patients with r-axSpA, cfDNA might also display its capacity to distinguish the inflammatory status. These results suggested that cfDNA might be a promising marker for r-axSpA activity.

Both DMARDs and TNFi contribute to the suppression of overactivated immune responses, while NSAIDs tend to reduce mediators of pain. In SLE and RA settings, DMARDs and TNFi could significantly diminish cfDNA^5,25–28^. As expected, cfDNA levels fell with treatment containing DMARDs and TNFi but not NSAIDs alone. Further analysis in subgroups, we found that responders to TNFi showed the most significant decrease in cfDNA after treatment. Consistently, TNFi were reported to lead to a dramatic decrease after 1 h infusion in RA patients^29^. Hashimoto et al. demonstrated that higher plasma cfDNA at 8 weeks can be a predictor of an early favorable response to DMARDs from 12 to 24 weeks^25^. Similarly, r-axSpA patients with high levels of cfDNA at diagnosis showed poor response to NSAIDs but could be reversed by combination with DMARDs or TNFi. Lower levels of cfDNA at diagnosis displayed an excellent response to NSAIDs. This may suggest that patients with high cfDNA levels at diagnosis may need to initiate more aggressive therapy including DMARDs or TNFi while patients with lower levels of cfDNA might benefit from mono-NSAID therapy.

There are some limitations in the current study. A small sample size might lead to bias in the results, especially in the role of cfDNA in predicting therapeutic response. More deliberately designed and larger cohorts are warranted to verify the results in future studies. Additionally, the mechanism of the effective efficacy of DMARDs and TNFi in patients with high cfDNA also remains unknown.

In conclusion, cfDNA may serve as a useful biomarker in reflecting disease activity and monitoring treatment response as well as optimizing the drug selection at diagnosis in r-axSpA.

## Data Availability

The experimental data will be available on request from the corresponding author.
